# Involvement of oxidative species in cyclosporine-mediated cholestasis

**DOI:** 10.3389/fphar.2022.1004844

**Published:** 2022-11-08

**Authors:** Bernard Nsengimana, Edozie Samuel Okpara, Wanqing Hou, Chuyun Yan, Shuxin Han

**Affiliations:** Department of Hepatobiliary Surgery, Anhui Province Key Laboratory of Hepatopancreatobiliary Surgery, The First Affiliated Hospital of USTC, Division of Life Sciences and Medicine, University of Science and Technology of China, Hefei, Anhui, China

**Keywords:** cyclosporine, cholestasis, oxidative stress, mitochondrial DNA, calcium

## Abstract

Cyclosporine is an established medication for the prevention of transplant rejection. However, adverse consequences such as nephrotoxicity, hepatotoxicity, and cholestasis have been associated with prolonged usage. In cyclosporine-induced obstructive and chronic cholestasis, for example, the overproduction of oxidative stress is significantly increased. Additionally, cyclosporine exerts adverse effects on liver function and redox balance responses in treated rats, as evidenced by its increasing levels of aspartate aminotransferase (AST), alanine aminotransferase (ALT), and bilirubin while also decreasing the levels of glutathione and NADPH. Cyclosporine binds to cyclophilin to produce its therapeutic effects, and the resulting complex inhibits calcineurin, causing calcium to accumulate in the mitochondria. Accumulating calcium with concomitant mitochondrial abnormalities induces oxidative stress, perturbation in ATP balance, and failure of calcium pumps. Also, cyclosporine-induced phagocyte oxidative stress generation *via* the interaction of phagocytes with Toll-like receptor-4 has been studied. The adverse effect of cyclosporine may be amplified by the release of mitochondrial DNA, mediated by oxidative stress-induced mitochondrial damage. Given the uncertainty surrounding the mechanism of cyclosporine-induced oxidative stress in cholestasis, we aim to illuminate the involvement of oxidative stress in cyclosporine-mediated cholestasis and also explore possible strategic interventions that may be applied in the future.

## Introduction

Cholestasis is a liver disease that causes a reduction or blockade in the flow of bile from the liver to the duodenum. Cholestasis can be intrahepatic or extrahepatic, with metabolic alterations, hepatitis, infection, and adverse drug reactions being the main causes of intrahepatic cholestasis and gallstones, cysts, and tumors being the main mechanical obstructions that lead to extrahepatic cholestasis. Because hydrophobic bile acids are harmful to hepatocytes and cholangiocytes, obstruction of bile flow causes retention of bile acids in the liver, resulting in liver damage (Nguyen et al., 2014; Visentin et al., 2018).

Individuals with prolonged severe intrahepatic cholestasis (PSIC) have a higher mortality risk (44%) than patients without PSIC (20%) after orthotopic liver transplantation (Fusai et al., 2006). Cyclosporine, a potential immunosuppressant drug used in organ transplantation, can mediate the induction of the cholestasis (Bluhm et al., 1992). By inducing liver damage, cyclosporine inhibits vesicle intrahepatic transporters and also competes with ATP-driven transporters (Kolaric et al., 2019). Notably, 50% of patients develop cholestasis following a liver transplant, and 12% of cholestatic patients experience cyclosporine toxicity (Pecorella et al., 1990). Cyclosporine-induced cholestasis can subsequently exaggerate the liver damage and may require retransplantation. The impact of immunosuppressive drugs (cyclosporine and tacrolimus) in post-transplant patients has been shown to induce hepatotoxicity and cholestasis (Taniai et al., 2008). Furthermore, cyclosporine induces cholestasis in animals by decreasing bile flow and bile salt output while also elevating bile cholesterol levels, a significant risk factor for gallstone formation (Chan and Shaffer, 1997). Another study on an isolated perfused rat liver indicates that at 150 mg/L concentration, cyclosporine severely inhibits bile flow and begins to induce a cholestasis (Deters et al., 1997). Moreover, as seen in HepaRG cells, higher cyclosporine concentrations (≥50 μM) are associated with irreversible alterations in efflux and uptake activities, which ultimately resulted in the constriction of bile canaliculi and disorganization of the pericanalicular F-actin microfilaments (Sharanek et al., 2014). Although cyclosporine modulates bile acids flow, most studies are being carried out on isolated perfused rat liver with a supratherapeutic concentration of cyclosporine. Future studies could document the impact of cyclosporine on bile acids based on *in vivo* studies. Essentially, the effectiveness of organ transplantation has been considerably influenced by the use of cyclosporine to prevent allograft rejection. However, this outstanding accomplishment has certain unfavorable consequences, such as hypertension, nephrotoxicity, and liver toxicity (Rezzani, 2004). Importantly, several distinct processes, such as inflammatory reactions, programmed cell death, autophagy, and reactive oxygen species (ROS), are involved in the cyclosporine-induced nephrotoxicity (Lai et al., 2017). Palomero et al. (2001) show that cyclosporine-treated rats exhibit high levels of oxidized glutathione (GSSH) and oxidative stress, reduced superoxide dismutase activity, reduced glutathione (GSH) levels, and induction of cholestatic features in treated rats. Thus, understanding the potential mechanism(s) underlying cyclosporine-induced hepatotoxicity and whether or not the overproduction of oxidative species is an integral driving factor is still a fascinating subject, to this review seeks to partly illuminate.

According to a previous study, the liver function of cyclosporine-treated rats was also reduced with a corollary increase in blood levels of AST, ALT, and bilirubin (Korolczuk et al., 2016). Treated animals showed low levels of GSH (3.3 nmol/g vs. 6.65 nmol/g) and high levels of GSSG (1.33 nmol/g vs 0.38 nmol/g) compared with the control. Unsurprisingly, the ratio of NADP/NADPH was found to be higher in treated rats than in the control (3.48 vs. 0.72), which underlines the role of oxidative stress as a key factor in the molecular etiology of cyclosporine-induced liver damage (Korolczuk et al., 2016). In addition, the effect of cyclosporine on cholestasis has been studied in 55 patients who underwent a kidney transplant and were treated with cyclosporine. Findings show that total serum biliary salts, total bilirubin, alkaline phosphatase, and biliary lithiasis are increased in transplant patients compared with the control group (Soresi et al., 1995).

In obstructive cholestasis, chronic cholestasis, and other hepatopathies, the establishment of oxidative stress is significantly increased (Copple et al., 2010). Wolf et al. demonstrated that treatment of rat hepatocytes in a dose-dependent manner generated large amounts of free reactive species while also attenuating the levels of glutathione and the antioxidant ascorbic acid (Wolf et al., 1997). After receiving cyclosporine treatment, reactive species, redox imbalance, and mitochondrial DNA damage, significantly increased in rat hepatocytes (Korolczuk et al., 2016). Concerning glutathione levels, rats receiving oral treatment with cyclosporine experienced decreases in the mRNA expression levels of glutathione synthesizing enzyme (gamma glutamylcysteine light chain and heavy chain), which in turn affected the bile flow (Bramow et al., 2001). These findings further elucidate the potential role of oxidative stress, as well as GSH depletion, as driving mechanisms in the development of the cyclosporine-induced cholestasis (Moran et al., 1998; Vickers et al., 2017). Although glutathione is regarded as the “super” cofactor of detoxifying enzymes and central antioxidants against oxygen species its role in cyclosporine-mediated cholestasis is still not fully understood. Determining how reactive species are involved and whether glutathione may have a protective impact in cyclosporine-induced cholestasis is the central aim of the current review.

### Cyclosporine: A potent inducer of oxidative species

Upon its identification in the fungus *Tolypocladium inflatum* by the Swiss pharmaceutical company Sandoz Ltd., in 1971, cyclosporine was regarded as a narrow-spectrum antibiotic and antifungal agent with very constrained clinical applications. However, following the establishment of its immunosuppressive properties on T-lymphocytes, cyclosporine is now approved for use in autoimmune diseases (rheumatoid arthritis, psoriasis, Crohn’s disease, and keratoconjunctivitis) and has also significantly benefited organ transplantation since 1983 (Fattizzo et al., 2022). Despite cyclosporine’s usefulness in limiting organ transplant rejection, its use has been associated with some negative side effects, such as nephrotoxicity, neurotoxicity, and hepatotoxicity. Hepatotoxicity is characterized by symptoms such as jaundice, weariness, loss of appetite, and weight loss (Yalcin et al., 2021). Also, hepatotoxicity due to cyclosporine use has been associated with increased blood levels of bilirubin, lactate dehydrogenase, alanine aminotransferase, and alkaline phosphatase. Consequently, liver cells begin to exhibit vacuolar degeneration, turbidity, hypertrophy, apoptosis, and nuclear deterioration in the early stages of cyclosporine-induced hepatotoxicity. Moreover, these characteristics are linked to central lobule structural degradation, loss of cord-like structure, and lymphocyte and neutrophil infiltration (Patocka et al., 2021). Indeed, variability in serum levels has been identified as a contributory factor in cyclosporine-induced toxicity, and significant cyclosporine variability has also been linked to a high likelihood of allograft recurrent rejection (Flippin et al., 2000).

Importantly, cyclosporine-induced hepatotoxicity is a complex process that includes elevated intracellular calcium levels, malfunctioning mitochondria, and the generation of free radicals in the liver. Although the high dose (15 mg/kg) was used to indicate the effect of cyclosporine treated rats, rather than the actual dose used for humans (2.5 mg/kg), results demonstrate that cyclosporine medication can harm liver function and cause oxidative stress and redox imbalances in rat hepatocytes (Korolczuk et al., 2016). These findings reveal that prolonged usage of cyclosporine may culminate in impaired redox stability, thereby, causing cholestasis. Additionally, findings from the same study provide additional evidence that the major driving mechanisms in cyclosporine-induced hepatotoxicity are mitochondrial damage and oxidative stress (Korolczuk et al., 2016). Notably, the buildup of ROS caused by cyclosporine-mediated liver injury increases hydrogen peroxide levels and decreases superoxide dismutase activity (Andres and Cascales, 2002). Furthermore, the induction of calcium accumulation in the mitochondria due to cyclosporine treatment has also been identified as a toxic mechanism, as mitochondrial calcium accumulation disrupts ATP synthesis and results in the failure of the membrane calcium pump (Salducci et al., 1996).

### The mechanism by which cyclosporine induces oxygen species

As a potent immunosuppressant, the major therapeutic effect of cyclosporine is the suppression of T-cell activity. T-cell receptors activate calcineurin by increasing intracellular calcium through calmodulin to elicit the T-cell response. The nuclear factor of activated T cells (NFAT) is then released from the cytosol by calcineurin, allowing for the entrance of NFAT into the nucleus, where it transactivates the expression of interleukin-2 and other cytokines (Fellman et al., 2019; Kitamura et al., 2020). Importantly, by attaching to the cytosolic protein cyclophilin D, cyclosporine stops this process. The cyclosporine-cyclophilin D complex then inhibits the action of the calcineurin enzyme, which in turn stops the generation of cytokines (Ke and Huai, 2004). Recent evidence from a yeast study reveals that a reduction in cytosolic calcium following the inhibition of the calcium/calcineurin pathway is associated with an increased level of ROS (Li et al., 2021). In a mammalian cell study, cyclosporine as a calcineurin inhibitor, promotes ROS formation, redox imbalance, and mitochondrial damage to induce hepatotoxicity (Korolczuk et al., 2016).

Interestingly, how cyclosporine contributes to hepatotoxicity caused by ROS is still a subject of open debate. ROS and NADPH oxidase-2 are produced in higher quantities in mice with calcineurin alpha knockdown, and cyclosporine-treated mice also exhibit similar outcomes (Cheriyan et al., 2021). However, cyclosporine can cause mitochondrial ROS accumulation without the involvement of the calcineurin (Zhou and Ryeom, 2014). By stimulating the NF-kB and toll-like receptor-4 (TLR4) pathways, *in vitro* cultures of murine primary cells study show that cyclosporine alone can sufficiently enhance the ROS generation (Rodrigues-Diez et al., 2016). Additionally, ROS are produced when TLR1, TLR2, and TLR4 stimulate the translocation of tumor necrosis factor receptor-associated factor 6 (TRAF6) into the mitochondria, promoting the degradation of the evolutionarily conserved signaling intermediate in the toll (ECSIT) protein (West et al., 2011).

**FIGURE 1:**
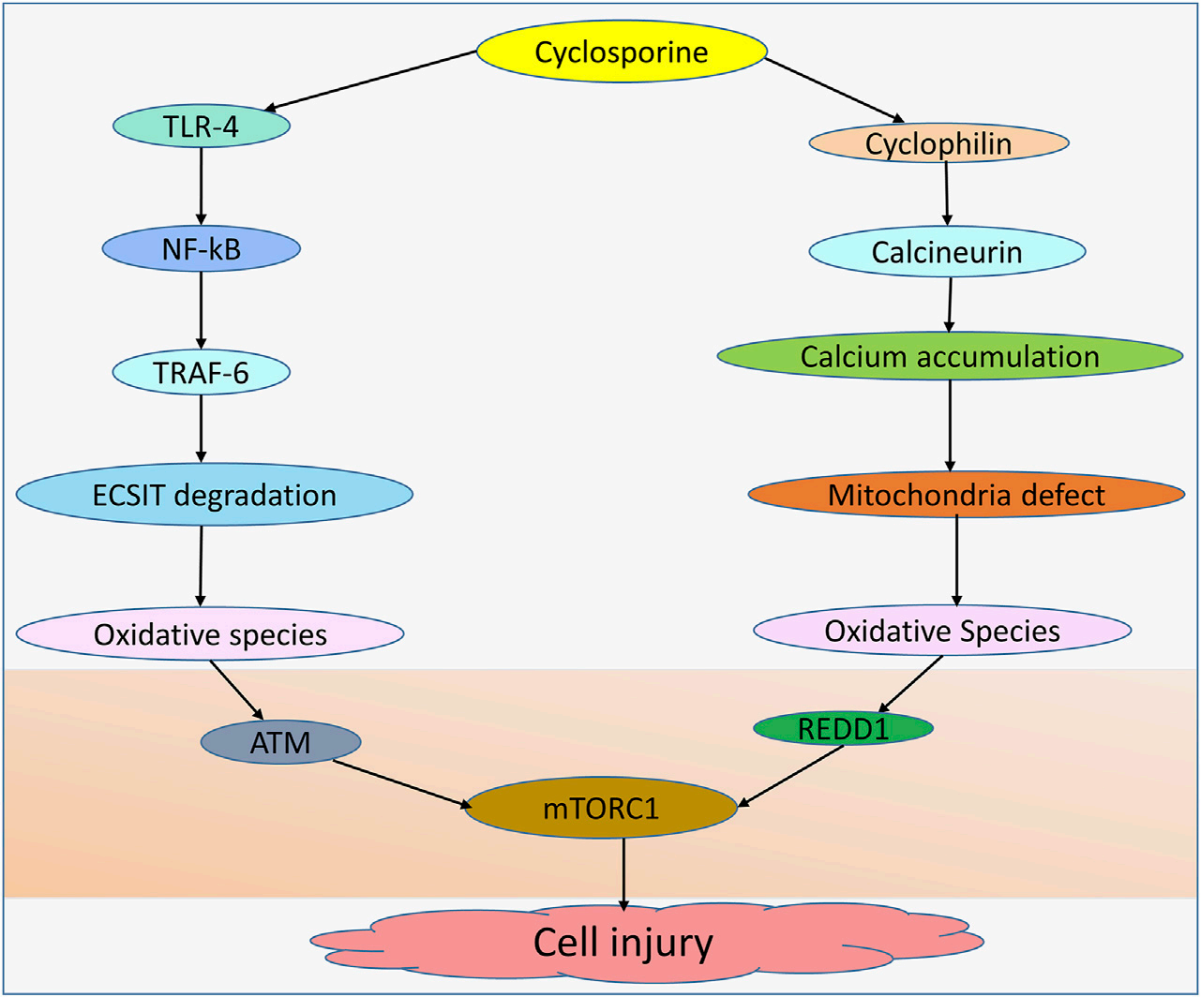
Cyclosporine-induced cell injury. Cell injury can be induced by cyclosporine through different mechanisms. Cyclosporine can interact with TLR-4 in the NF-kB pathway, allowing TRAF-6 to translocate into the nucleus and induce ECSIT degradation, leading to the production of oxidative species. Cyclosporine-cyclophilin can mediate mitochondrial dysfunction due to the accumulation of calcium deposits, leading to oxidative species generation. Oxidative species inhibit the expression of mTORC1 by upregulating ATM and REDD1, resulting in cell injury.

Although hepatocytes isolated from cyclophilin D knockdown resist calcium overload and oxidative stress-induced cell death (Baines et al., 2005) and cyclosporine inhibits oxidation rates by hindering mitochondrial calcium efflux (Fournier et al., 1987), mitochondrial permeability transition pore (MPTP) can be mediated by other factors, including calcium, ROS, and lipid pores, allowing for MPTP-stimulated generation of ROS and induction of caspase-3 activity, which ultimately results in cell damage (Mironova and Pavlov, 2021; Skinner et al., 2021). The evidence further shows that cyclosporine can induce toxicity and this is dependent on the concentration used by mediating mitochondrial alterations (Jung and Pergande, 1985). Moreover, glycochenodeoxycholic acid increases the production of ROS in the mitochondria of human hepatocytes, a process essential for the induction of MPTP in a dose-dependent manner (Sokol et al., 2005). Notably, an interaction between cyclosporine and cyclophilin D has been linked to mitochondrial dysfunction and ROS generation (Klawitter et al., 2019). Hence, in wild-type mice treated with cyclosporine but not in cyclophilin D mutant animals, oxidative stress and 8-isoprostane levels are increased (Klawitter et al., 2019). To evaluate the involvement of MPTP in cholestasis, prior work in a mouse model showed that casein can enter the mitochondria following bile duct ligation (BDL), demonstrating the pathogenic role of MPTP in the cholestasis (Rehman et al., 2008). Despite the evidence demonstrating cyclosporine as a potent inhibitor of calcium-dependent MPTP in rat liver (Broekemeier et al., 1989), cyclosporine may also compromise the function of mitochondria and cause reactive species generation through a couple of other distinct routes (Figure 1).

### Mitochondrial dysfunction and oxidative species

Cholestasis is characterized by the induction of oxidative stress and a dysfunctional mitochondrial architecture (Vinken, 2015). In contrast to untreated cholestatic animals, rats treated with edaravone following BDL showed elevated levels of ROS, oxidized glutathione, mitochondrial depolarization and permeability, reduced dehydrogenase activity, and fibrotic lesions (Mohammad Mehdi et al., 2021).

Patients with cholestatic disease frequently present increased expression of mitochondrial methylation-controlled J-protein (MCJ), which inhibits electron transport chain complex I. For this reason, bile acid-induced mitochondrial ROS upregulation is scarce in MCJ-knockout mice (Iruzubieta et al., 2021). Rats subjected to BDL showed a decrease in mitochondrial biogenesis transcriptional regulators, such as liver peroxisome proliferator-activated receptor gamma coactivator-1alpha and mitochondrial transcription factor A, which demonstrates the role of mitochondrial dysfunction in cholestasis. Moreover, mitochondrial DNA (mtDNA) copies are also decreased to the lowest levels after 72 h of BDL (Tiao et al., 2009). Damage to the mitochondria can increase the severity of the disease by generating ROS, which then causes an accumulation of cytotoxic chemicals and ATP depletion (Heidari and Niknahad, 2019). The findings further demonstrate that the mitochondrial bioenergetics of cholestatic individuals are altered by varying bile acid concentrations (Rolo et al., 2000).

It is well known that mitochondria in the majority of cells are the primary producers of reactive species. The respiratory chain I and III complex and the pathway for the oxidation of fatty acids in mitochondria are the main sources of ROS in these organelles. Importantly, glutathione peroxidase and manganese superoxide dismutase in the mitochondria normally prevent the buildup of the ROS (Garcia-Ruiz and Fernandez-Checa, 2018). However, a high level of ROS can result in mtDNA depletion as well as damage to mitochondrial elements, including cardiolipin and other proteins required for the efficient functioning of the mitochondrial respiratory chain. ROS has a significant impact on mtDNA, and mtDNA abnormalities, in turn, result in mitochondrial dysfunction. Numerous studies have demonstrated evidence of oxidative stress-induced mitochondrial abnormalities involved in cholestasis and a potential mechanism based on the altered mtDNA copy number and imbalance of oxidants *versus* antioxidants in the mitochondria (Iruzubieta et al., 2021).

The Cambridge reference sequence (CRS) is the first full sequence of human mitochondrial DNA and was released in 1981. Led by Fred Sanger, the CRS project, which involved the sequencing of mtDNA from women of European heritage in the 1970s, results in greater insights into the evolution of human mitochondrial diseases (Andrews et al., 1999). Within each mitochondrion, there are many copies of circular, double-stranded human mtDNA, which are 16.6 kb each in size. By encoding the 13 polypeptides essential for oxidative phosphorylation, two ribosomal RNAs, and 22 transfer RNAs required for mitochondrial translation, mtDNA controls the efficiency of the mitochondrial respiratory chain and ATP generation. In addition to the protective effect of histones on DNA, mtDNA is safeguarded by mitochondrial transfer factor A (TFAM), DNA polymerase gamma, and mitochondrial single-stranded DNA-binding protein (mtSSB) (Zhao et al., 2021). Assessment of how oxidative stress affects mtDNA in liver injury, especially in cholestasis, is of crucial importance because more mtDNA in hepatocytes and other liver cells is associated with a high number of mitochondria (approximately 1,000–2,000) than in white cells, which have approximately 30–100 mitochondria.

Importantly, to start the mitochondrial functional deficiency, mtDNA copies must fall below 20%–40% of the basal line. Cholestatic patients exhibit a high level of mitochondrial 8-hydroxydeoxyguanosine and deficient mtDNA characteristics, such as copy number, transcript level, and nucleoid structure, which are used to determine the alteration of mtDNA copies during cholestatic liver injury (Xu et al., 2012). The impact of mitochondrial failure on several biological processes, such as inflammatory stress, metabolic disorders, oxidative stress, and fibrosis, has been intensively studied as being related to cholestasis. Notably, cholestasis can become more severe when there are both mitochondrial abnormalities and mtDNA depletion or deletion (Arduini et al., 2012). The aforementioned data indicate that oxidative stress plays a significant role in mitochondrial dysfunction and hepatocyte damage. A recent study showed that oxidative phosphorylation-produced ROS can cause mtDNA damage (Nissanka and Moraes, 2018). Additionally, numerous diseases are linked to oxidative stress-induced mtDNA damage (Huang et al., 2020). Similarly, a mouse model study showed that a reduction in mitochondrial DNA polymerase g (POLG) is linked to higher levels of mutations and more apoptotic markers relative to the control group with intact POLG expression levels (Lee and Wei, 1997; Kujoth et al., 2005). In addition, patients with liver diseases have reduced expression levels of mtDNA-encoded proteins (Santamaria et al., 2003). Liver damage is promoted by a decrease in mtDNA in liver cells, which obstructs mitochondrial function (Demeilliers et al., 2002).

As mtDNA research is still in its infancy, few studies have yet shown that the impact of several drugs on hepatotoxicity is based on interrupting mtDNA activity. The drugs can induce mitochondrial toxicity by inhibiting mtDNA replication, translation, and/or methylation, as summarized in Table 1 (Le Guillou et al., 2018; Fromenty, 2020). Hence, mtDNA mitochondriotoxicity is expected to be a potentially ‘hot’ research direction for drug-induced liver toxicity. To the best of our knowledge, there is currently no record of any research that has examined the effects of cyclosporine on mtDNA mitochondriototoxicity-induced liver injury; however, only two studies have examined its effects on lung injury in the context of inflammation. According to the first study, cyclosporine inhibits inflammatory responses and mtDNA in the treated group after 15 min (Xiao et al., 2018). The second study indicates that mtDNA can act as a damage-associated molecular marker in lung injury. Only after the administration of cyclosporine for 15 min was a dose-dependent reduction in mtDNA and inflammatory responses observed (Liu et al., 2019). In sum, the integrity of mitochondria is crucial for cells, and the effects of medications on mitochondria as well as mtDNA are therefore used by pharmaceutical companies to evaluate the safety of drugs. It is also known that mitochondria play a tight regulatory role in calcium buffering. The relationship between mitochondria-mediated calcium homeostasis and oxidative stress-induced cholestasis remains to be further explored.

**TABLE 1:** Effect of some drugs on mtDNA.

Drug	Mode of action	Effect to mtDNA	References
4-quinolone	A fluoroquinolone which inhibits bacterial DNA topoisomerase during DNA replication	Decreases mtDNA content and reduces the mitochondrial respiration	Lawrence et al. (1993)
Fialuridine	1-(2-deoxy-2-fluoro-β-d-arabinofuranosyl)-5-iodouracil that used to treat chronic hepatitis B	Suppresses DNA polymerase gamma and promotes mtDNA reduction	Lewis et al. (1996)
Ganciclovir	9-(1,3-dihydroxy-2-propoxymethyl) guanine which inhibits viral DNA replication by targeting ganciclovir-5-triphosphate	mtDNA depletion and mitochondrial impairment	Herraiz et al. (2003)
Tacrine	It induced a reversible inhibition of acetylcholinesterase	Reduces the integration of [(3)H] thymidine into mtDNA, Reduces mtDNA, and partly unwound supercoiled mtDNA into circular mtDNA	Mansouri et al. (2003)
Tamoxifen	An anti-estrogenic drug that competes with 17β-estradiol at the receptor site and to block the activity of E_2_ in breast cancer	It inhibits topoisomerases and leads to mtDNA depletion	Larosche et al. (2007)
Linezolid	It inhibits bacterial protein synthesis by preventing the formation of a functional 70S initiation complex	It decreases the mtDNA-encoded peptide translation	De Vriese et al. (2006)
Tetracycline	Inhibits the protein synthesis by binding to aminoacyl tRNA to the A-site of the ribosome	It suppresses the mitochondrial translation	Skrtic et al. (2011)
Valproic Acid	An antiepileptic drug which binds on gamma aminobutyric acid	It promotes mtDNA methylation	Wolters et al. (2017)

According to the aforementioned studies, it is clear that mitochondrial abnormalities contribute to the onset of cholestasis, albeit the prevailing lack of clarity on the effect of cyclosporine on mitochondrial activity in cholestasis. Findings also reveal that the liver function examination of a rat treated with cyclosporine exhibits significant changes in oxidative stress markers, a considerable rise in ALT, AST, and bilirubin levels in serum, mononuclear cell infiltration, and obvious mitochondrial damage (Korolczuk et al., 2016; Dada et al., 2021). Depending on the dosage used, cyclosporine can also protect mitochondria by suppressing MPTP (Almofti et al., 2003; Hicks et al., 2009). Ions with sizes less than 1,500 Da are known to enter the MPTP. The enlargement and breakdown of the mitochondrial membrane potential are linked to this buildup of chemicals inside the mitochondria. To prevent hepatocyte necrosis, cyclosporine and trifluoperazine block the pores (Broekemeier et al., 1989; Imberti et al., 1993). A recent study, however, found that cyclosporine can, in an age-dependent manner, extend the time needed to induce MPTP. Furthermore, MPTP can be induced by bile acids, particularly glycochenodeoxycholate, which is one of the bile salts whose concentration rises by more than 20-fold during cholestasis (Yerushalmi et al., 2001). Interestingly, the combination of trifluoperazine and cyclosporine prevents the opening of the mitochondrial permeability pore caused by the glycochenodeoxycholate (Gores et al., 1998). Taken together, apart from being an inducer of oxidative species, cyclosporine can inhibit cytosolic calcium, and the evidence further shows that aberrant cytosolic calcium and high accumulation of calcium in mitochondria are also associated with ROS formation. However, mitochondrial defects are associated with cholestasis, and the combination of cyclosporine and trifluoperazine can suppress MPTP. Future studies could clarify to what extent cyclosporine can induce mitochondrial damage or protect the MPTP.

### Endoplasmic reticulum-calcium signaling-mitochondrial damage crosstalk

Calcium is a multipurpose second messenger that regulates a wide range of physiological pathways, including lipid and glucose metabolism, cell replication, cell death, and bile secretion (Amaya and Nathanson, 2013). Liver damage may correlate with the dysregulation of the calcium homeostasis (Oliva-Vilarnau et al., 2018). Although it is well known that calcium is mostly deposited in the endoplasmic reticulum, calcium signaling controls mitochondrial function, particularly *via* the regulation of Krebs cycle enzymes; mitochondria, also controls the calcium dynamicity (Bravo-Sagua et al., 2017). Depending on the inositol 1,4,5-triphosphate receptor (IP3R) and the chaperone protein glucose-regulated protein 75 (GRP75), calcium could gain entrance into the mitochondria through a mitochondria-associated membrane (MAM), which then triggers the opening of the mitochondrial calcium unidirectional transporter (MCU) (Wang et al., 2020). Furthermore, a sodium-calcium exchanger is used by liver cells to absorb 60% of the calcium (Bernstein and Santacana, 1985).

A more recent study confirmed that mtDNA can impact calcium signaling, stressing that cells with mtDNA depletion exhibit significantly reduced calcium signaling cascades (Sherer et al., 2000). In a similar study, mtDNA polymorphism analysis demonstrated that transmitochondrial cytoplasmic hybrid cells with 8701A/10398A are more closely related to the intracellular calcium dynamicity (Kazuno et al., 2006). However, another study showed that 25 μg/ml of ciprofloxacin can cause cells to lose 60% mtDNA content, which therefore results in a decreased mitochondrial membrane potential and mitochondria-mediated calcium buffering capacity. Incubation with ciprofloxacin for 4 and 11 days reduces the cellular calcium entry rate by 33% and 50%, respectively (Koziel et al., 2006).

### Calcium signaling and related biological processes

Although our focus in the current review is on oxidative stress and cholestasis, we have also summarized the effect of calcium on related biological processes, especially the endoplasmic reticulum. According to a recent study, the opening of the sodium-calcium exchanger affects the hydrophobic bile acid activity (Zhu et al., 2016). Although calcium controls Krebs cycle enzymes in mitochondria, research suggests that sodium-calcium exchangers also control oxidative stress. Increased calcium input into mitochondria and production of ROS are observed when the expression of the sodium-calcium exchanger is knocked down in rats using siRNA (Zu et al., 2015). A similar phenomenon occurs in rabbit cardiomyocytes, where calcium entry *via* a sodium-calcium exchanger results in ROS and cardiac damage (Wagner et al., 2003). The study demonstrated that reconfiguration of MAM is linked to excessive calcium accumulation, decreased oxidative capacity, and increased oxygen reactive species in the mitochondria (Arruda et al., 2014).

Furthermore, cholestasis is characterized by the IP3R loss (Shibao et al., 2003). In cholestatic individuals, IP3R is inhibited by miRNA-506, which targets its 3′-UTR and blocks the calcium transmission (Ananthanarayanan et al., 2015). These findings demonstrate the involvement of calcium transporters in calcium-induced oxidative species and the aberrant expression of these transporters in cholestasis. Additionally, pretreatment phenylephrine hepatocytes exposed to a hazardous amount of menadione increase the quantity of calcium in the cytosol, demonstrating the relationship between oxidative stress and the calcium signaling (Nicotera et al., 1988). ROS can be produced by mitochondria in a variety of ways, including by accelerating metabolic reaction rates, causing the release of cytochrome c and MPTP, and by activating the calcium-calmodulin transmission (Peng and Jou, 2010). In addition, 10 mM H_2_O_2_ was employed to examine how oxidative species affected calcium signaling. The findings demonstrate that the dosage of H_2_O_2_ causes a rise in mitochondrial calcium while having no effect on the cytoplasmic calcium (Greene et al., 2002).

Importantly, the production of ROS is crucial to understanding the overall effect of ROS generation on endoplasmic reticulum stress. ROS generated due to the entry of calcium into the mitochondria, can induce endoplasmic reticulum stress under conditions of liver injury, especially drug-induced liver toxicity (Zeeshan et al., 2016). The overproduction of ROS compromises the functional integrity of the endoplasmic reticulum for calcium release, allowing for the induction of cholestasis. A fatal hypermetabolic reaction is linked to low expression of calsequestrin-1, a calcium-binding protein in the sarcoplasmic reticulum. In animals given the antioxidant N-acetylcysteine, calsequestrin-1 knockout reduced mitochondrial superoxide production while increasing glutathione levels in comparison to the control group (Michelucci et al., 2015). Importantly, the abnormal expression of calsequestrin-1 is linked to high levels of oxidative stress production and mitochondrial damage (Paolini et al., 2015). Furthermore, the inhibition of GRP75 reduces the buildup of calcium in the mitochondria and the production of ROS (Honrath et al., 2017).

MPTP impairment is promoted by aberrant mitochondrial calcium levels caused by naphthylisothiocyanate, a cholestasis-inducing agent (Reichen et al., 1985). Calcium deficiency causes cholestasis by increasing biliary permeability and canalicular dysfunction. Although mitochondria and the endoplasmic reticulum are linked to calcium homeostasis, endoplasmic calcium is not the inducer of the cholestasis (Farrell et al., 1990). However, biliary epithelial cells have higher levels of endoplasmic reticulum stress markers, which drive cell death (Sasaki et al., 2015). As oxidative stress can be controlled by the endoplasmic reticulum, tunicamycin, an endoplasmic reticulum stressor, also causes oxidative stress, elevated levels of AST and ALT, and liver cell damage due to lipid peroxidation and glutathione depletion. Curiously, therapy with 2% taurine reverses these effects (Kim et al., 2022). The link between endoplasmic reticulum stress and drug-induced cholestasis has also been demonstrated. The results show that liver injury precedes the generation of ROS caused by endoplasmic reticulum stress, which is an early event in drug toxicity-mediated cholestasis. Essentially, the activation of HSP27-PI3K-AKT decreases drug-induced endoplasmic reticulum stress as well as oxidative stress and also abrogates the liver damage (Burban et al., 2018).

### Mechanisms of cyclosporine-induced cell injury

#### Induction of endoplasmic reticulum stress

**FIGURE 2:**
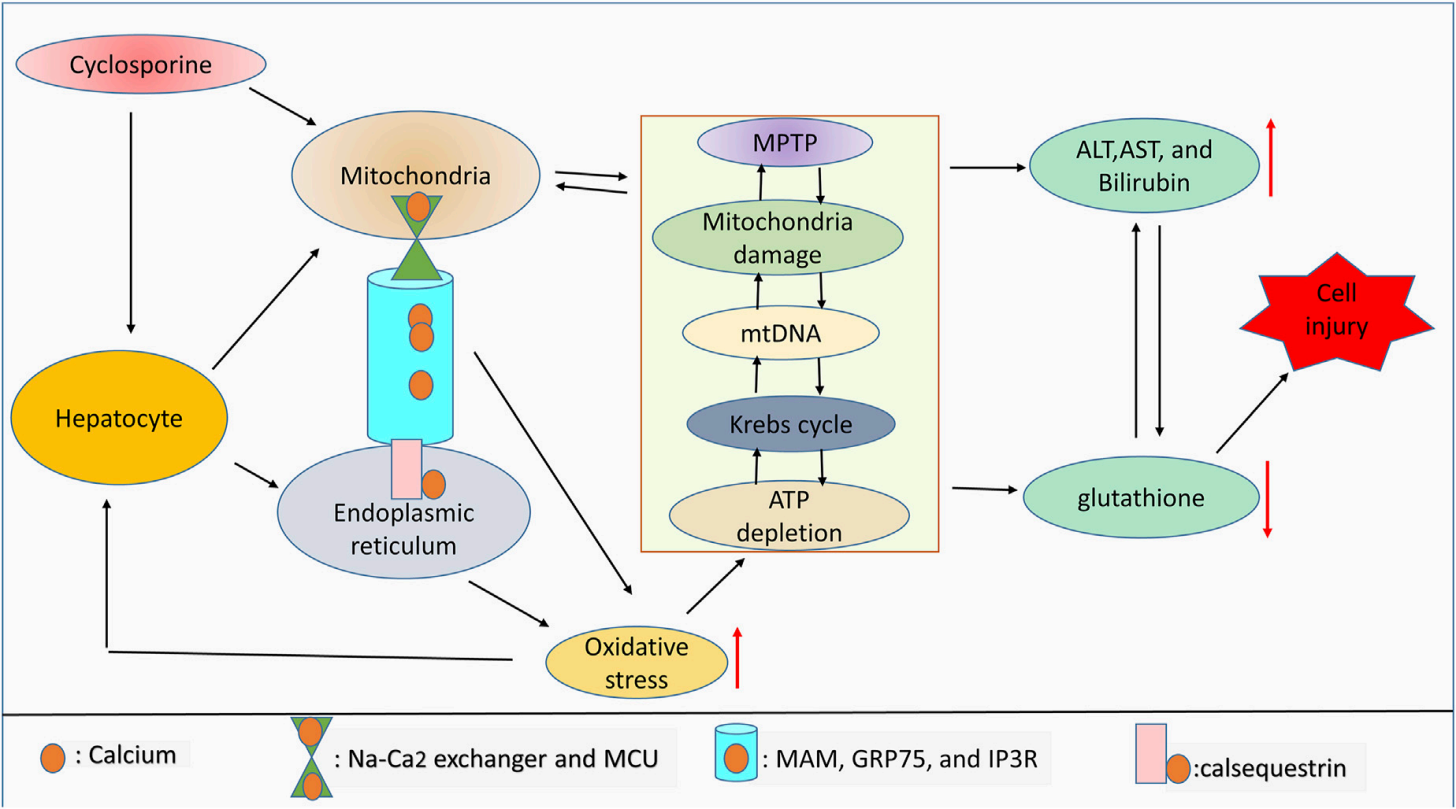
ROS and interorganelle communication. Notably, calsequestrin regulates calcium dynamicity inside the endoplasmic reticulum, whereas MAM, GRP75, and IP3R regulate the movement of calcium from the endoplasmic reticulum into mitochondria. Inside mitochondria, calcium movement is controlled by the Na-Ca^2^ exchanger and MCU. Dysregulation of this endoplasmic reticulum-mitochondria collaboration due to drug toxicity can lead to oxidative stress generation. Aberrant calcium signaling and high levels of ROS can affect mitochondrial integrity, mitochondrial function, and ATP production. Mitochondrial damage and high levels of ROS are associated with antioxidant depletion and limited liver function, which leads to cell injury. Therefore, Mitochondrial damage will indirectly correlate with the increase of liver cell injury markers including ALT, AST, and bilirubin due to the drug toxicity-induced high ROS accumulation.

Cyclosporine has been shown to mediate chronic nephrotoxicity. Results from the literature review show that cyclosporine produces endoplasmic reticulum stress, which results in protomyofibroblast formation and activation of transforming growth factor. Interestingly, salubrinal, which lessens endoplasmic reticulum stress, protects against phenotypic changes in epithelial cells (Pallet et al., 2008). Furthermore, cyclosporine increases endoplasmic reticulum stress by increasing GRP78 expression and stimulating the apoptosis (Liu et al., 2015). It is crucial to determine whether endoplasmic reticulum stress and oxidative stress are connected with cyclosporine use in cholestasis. In a concentration-dependent manner, cyclosporine has been shown to induce endoplasmic reticulum stress in HepaRG cell lines, which is followed by the generation of oxidative stress. These alterations are related to aberrant expression of the bile acid enzyme regulator and bile acid buildup in cells and culture media (Sharanek et al., 2014; Gijbels et al., 2020). Contrastively, the findings show that, depending on the level of calcium, cyclosporine can protect hepatocytes against oxidative stress. Notably, cyclosporine protects cells treated with 0.8 mM t-butylhydroperoxide with 10 mM calcium, but this protection is ineffective at 2.5 mM calcium (Broekemeier et al., 1992). Furthermore, while infantile cholestasis caused by toxic bile acids results in liver damage and mortality, the role of calcium signaling in this pathophysiology is still unknown. Specifically, calcium-sensing receptors are elevated during infantile cholestasis, and their elevation enhances the activation of p-extracellular signal-regulated kinase (p-ERK) which in turn lessens the expression of apoptotic markers, lowers intracellular calcium, and also inhibits the development of oxidative stress (Qin et al., 2020). As summarized in Figure 2, cyclosporine can induce cell injury by mediating ROS generation due to interorganelle communication.

#### Reactive oxygen species involvement and the role of reduced glutathione

Glutathione is generated by the formation of a gamma peptide bond between glutamate and cysteine, allowing for the binding of glycine to the carboxyl group of glutamylcysteine. As an antioxidant, glutathione has been shown to not only protect cells from ROS, such as peroxidase and free radicals but it has also been shown to be the central positive regulator of the cell’s antioxidant systems (Forman et al., 2009). The biosynthesis of glutathione involves two stages, the first of which is catalyzed by glutamate-cysteine ligase/glutamate cysteine synthase and involves the conversion of L-glutamate and cysteine into gamma-glutamylcysteine a precursor of glutathione. In stage 2 of this process, C-terminal gamma-glutamylcysteine receives glycine from glutathione synthase, allowing for the generation of GSH. As a cofactor of glutathione peroxidase and as part of the liver detoxification and biotransformation processes, glutathione participates in a variety of cellular processes (Pastore et al., 2003). Glutathione reductase catalyzes the conversion of glutathione disulfide into its reduced state, a process requiring NADPH. GSH thereafter protects cells by neutralizing ROS and free radicals (Xiao and Loscalzo, 2020).

As part of their defense system, normal liver cells typically release minute amounts of ROS. Moreover, mitochondria could also be an intracellular source of ROS since complexes I and III are capable of releasing electrons. Importantly, hepatocytes have developed defenses to mitigate the detrimental buildup of ROS within the cell, and GSH-coordinated antioxidant systems, such as catalase, glutathione peroxidase, and superoxide dismutase, play essential roles in this regard (Cichoz-Lach and Michalak, 2014). Generally, hepatocytes maintain redox-active iron for protein localization and possess a layered defense mechanism against ROS, which includes the presence of glutathione in all cellular compartments and vitamin C (an antioxidant) in cell membranes (Akbay et al., 2019). However, as glutathione levels drop in the mitochondria, ROS buildup precipitates, eventually causing the opening of the MPTP and the subsequent release of pro-apoptotic factors, principally endonuclease G, into the nucleus, where they cause DNA fragmentation and ATP depletion (Imaizumi and Aniya, 2011).

ROS generated by inflammation can also cause liver injury in addition to cell death caused by mitochondrial malfunction. Typically, cholestasis induces the molecular patterns linked with damage that cause the activation of the complement cascade (Jaeschke and Ramachandran, 2011). Cholestasis can also induce the activation of the TLR, which attracts neutrophil-like macrophages. ROS activation from phagocytic cells can be further aided by complement cascade activation and inflammatory response activation (Yang et al., 2019; Xiao and Loscalzo, 2020).

Mechanistically, to induce liver injury, a high concentration of ROS suppresses the effect of mammalian target of rapamycin complex 1 (mTORC1) by activating cytoplasmic ataxia telangiectasia mutated (ATM). ROS burst-induced peroxide enhances protein-1 activation, which then upregulates REDD1 and downregulates mTORC1. In addition, studies show that ROS regulates different cellular pathways, such as the AKT/mTOR, Notch, Wnt, and JAK-STAT pathways (Stacy Grieve, 2022). Similarly, ROS suppresses mTORC1 expression by upregulating AMPK. In addition, animal study shows that cyclosporine induces hepatic GSH depletion, while renal GSH remains unaffected (Jimenez et al., 2000). Interestingly, N-acetylcysteine, acting as an antioxidant, reinstates the mTORC1 activity (Zhang T. et al., 2019).

## Conclusion and future perspectives

Allograft rejection is significantly reduced by the use of cyclosporine. However, as a result of extended exposure or high serum levels of cyclosporine, patients may develop side effects such as hepatotoxicity and cholestasis. Therefore, alternative ways to improve the clinical outcome of this outstanding drug are urgently needed. Importantly, cyclosporine-induced cellular damage occurs *via* different key mechanisms: induction of mitochondrial calcium accumulation (which results in mitochondrial abnormalities and disruption in ATP homeostasis); ROS generation; and the translocation of TRAF-6 into the nucleus (following the interaction of cyclosporine with TRAF-4), which causes TRAF-6-induced ECSIT breakdown and fosters the formation of oxidative species. To elicit their effect, ROS modulate several pathways, including mTORC1, AKT/mTOR, Notch, Wnt, and JAK-STAT. In this regard, the potential function of antioxidants, such as glutathione, vitamin C, and N-acetylcysteine, in attenuating cyclosporine-induced hepatotoxicity, particularly cholestasis, can be used as strategic support. In an animal model of the disease, the antioxidant resveratrol demonstrates a protective potential against hepatotoxicity by attenuating cyclosporine-induced ROS accumulation, decreasing advanced oxidation protein products as well as suppressing levels of thiobarbituric acid reactive substances (Bingul et al., 2021).

Exploration of the role of cyclosporine in liver damage and cholestasis, in particular, based on the drug’s toxic effects on mitochondria, including mtDNA depletion, mitochondria-peptide translation impairment, and mtDNA methylation, is indeed an intriguing research interest. Notably, while mtDNA release causes the interconnection of both oxidative stress and inflammatory response formation, the inflammatory response generated is intensified as mtDNA release continues unabated, eventually altering the functional integrity of the mitochondria. Moreover, mtDNA release is regarded as a damage-associated molecular pattern in the context of inflammation that can activate TLRs and trigger the production of interferon and cytokines, leading to cell injury (Zhang X. et al., 2019). On the other hand, ROS can promote the activation of transcription factors connected to inflammatory signaling pathways (Reuter et al., 2010). Cell damage and chronic inflammation can be induced by the interaction between oxidative species and mitochondrial defect/mtDNA release. In addition, impaired protein translation is connected to drug-mediated mtDNA depletion. Therefore, a promising treatment strategy for liver damage and cholestasis could be a medication, especially those that increase serum levels of GSH that can improve oxidative pathways and also restore mitochondrial integrity.

It is worth noting that calcium signaling plays a vital role in the control of oxidative species in light of the aforementioned facts. Evidence also shows that endoplasmic reticulum-mitochondrial communication mediates the control of calcium signaling. By focusing on MAM and other calcium transporters, such as MCU, future studies can examine the usefulness of mitochondria-endoplasmic reticulum involvement in cholestasis. In addition, according to a recent study, more than 50% of cholestatic patients had low bone mineral density, and more than 50% of cholestatic patients had low vitamin D levels, which are important for calcium absorption (Samra et al., 2018). Interestingly, taking 25-hydroxyvitamin D3 orally can enhance calcium absorption (Bengoa et al., 1984). Therefore, it is crucial to compile more translational methods that may be applied in the future to improve calcium absorption and its effectiveness in cholestatic patients, as well as to effectively abrogate oxidative species and GSH plays an important role in this regard which has been observed to make integral contributions to the molecular mechanisms driving drug-induced toxicities.

By using different gene set collections such as Tox action, Kyoto Encyclopedia of Genes and Genomes (KEGG), and gene ontology (GO) Biol process, findings show that cyclosporine affects several biological processes including apoptosis, DNA repair, oxidative phosphorylation, endoplasmic reticulum stress, and oxidative stress (Schmeits et al., 2015). Moreover, the aforementioned studies reveal that cyclosporine modulates bile acids secretion, and bile acids accumulation impairs mitochondria bioenergetics. However, antioxidant usage inhibits bile acids-induce MPTP, which reveals that bile acids can alter the mitochondria activity by inducing ROS production. Therefore, mitochondrial integrity could clinically be taken into account, especially in cholestatic patients.

## Abbreviations

ALT, alanine aminotransferase; AST, aspartate aminotransferase; BDL, bile duct ligation; CRS, Cambridge Reference Sequence; ECSIT, Evolutionarily Conserved Signaling Intermediate in Toll pathway; GRP75, chaperone Glucose-Regulated Protein 75; GO, gene ontology; IP3R, inositol 1,4,5-triphosphate receptor; KEGG, Kyoto Encyclopedia of Genes and Genomes; mTORC1, mammalian target of rapamycin complex 1; MAM, Mitochondria-associated membrane; MCJ, Methylation-Controlled J-protein; MCU, Mitochondrial calcium unidirectional transporter; MPTP, Mitochondrial permeability transition pore; mtDNA, mitochondrial DNA; mtSSB, mitochondrial single-stranded DNA-binding protein; p-ERK, p-Extracellular Signal-Regulated Kinase; POLG, Mitochondrial DNA Polymerase G; PSIC, prolonged severe intrahepatic cholestasis; ROS, reactive oxygen species; TFAM, Mitochondria Transfer Factor A; TLR-4, Toll-like receptor-4; TRAF-6, Tumor necrosis factor-associated factor-6.

## References

[B1] Akbay E. Erdem B. Unlu A. Durukan A. B. Onur M. A. (2019). Effects of N-acetyl cysteine, vitamin E and vitamin C on liver glutathione levels following amiodarone treatment in rats. Kardiochir Torakochirurgia Pol. 16 (2), 88–92. 10.5114/kitp.2019.86361 31410096 PMC6690152

[B2] Almofti M. R. Ichikawa T. Yamashita K. Terada H. Shinohara Y. (2003). Silver ion induces a cyclosporine a-insensitive permeability transition in rat liver mitochondria and release of apoptogenic cytochrome C. J. Biochem. 134 (1), 43–49. 10.1093/jb/mvg111 12944369

[B3] Amaya M. J. Nathanson M. H. (2013). Calcium signaling in the liver. Compr. Physiol. 3 (1), 515–539. 10.1002/cphy.c120013 23720295 PMC3986042

[B4] Ananthanarayanan M. Banales J. M. Guerra M. T. Spirli C. Munoz-Garrido P. Mitchell-Richards K. (2015). Post-translational regulation of the type III inositol 1, 4, 5-trisphosphate receptor by miRNA-506. J. Biol. Chem. 290 (1), 184–196. 10.1074/jbc.M114.587030 25378392 PMC4281721

[B5] Andres D. Cascales M. (2002). Novel mechanism of Vitamin E protection against cyclosporine A cytotoxicity in cultured rat hepatocytes. Biochem. Pharmacol. 64 (2), 267–276. 10.1016/s0006-2952(02)01112-7 12123747

[B6] Andrews R. M. Kubacka I. Chinnery P. F. Lightowlers R. N. Turnbull D. M. Howell N. (1999). Reanalysis and revision of the Cambridge reference sequence for human mitochondrial DNA. Nat. Genet. 23 (2), 147. 10.1038/13779 10508508

[B7] Arduini A. Serviddio G. Tormos A. M. Monsalve M. Sastre J. (2012). Mitochondrial dysfunction in cholestatic liver diseases. Front. Biosci. 4 (6), 2233–2252. 10.2741/539 22202034

[B8] Arruda A. P. Pers B. M. Parlakgul G. Guney E. Inouye K. Hotamisligil G. S. (2014). Chronic enrichment of hepatic endoplasmic reticulum-mitochondria contact leads to mitochondrial dysfunction in obesity. Nat. Med. 20 (12), 1427–1435. 10.1038/nm.3735 25419710 PMC4412031

[B9] Baines C. P. Kaiser R. A. Purcell N. H. Blair N. S. Osinska H. Hambleton M. A. (2005). Loss of cyclophilin D reveals a critical role for mitochondrial permeability transition in cell death. Nature 434 (7033), 658–662. 10.1038/nature03434 15800627

[B10] Bengoa J. M. Sitrin M. D. Meredith S. Kelly S. E. Shah N. Baker A. L. (1984). Intestinal calcium absorption and vitamin D status in chronic cholestatic liver disease. Hepatology 4 (2), 261–265. 10.1002/hep.1840040215 6706300

[B11] Bernstein J. Santacana G. (1985). The Na+/Ca2+ exchange system of the liver cell. Res. Commun. Chem. Pathol. Pharmacol. 47 (1), 3–34. 2580336

[B12] Bingul I. Olgac V. Bekpinar S. Uysal M. (2021). The protective effect of resveratrol against cyclosporine A-induced oxidative stress and hepatotoxicity. Arch. Physiol. Biochem. 127 (6), 551–556. 10.1080/13813455.2019.1659826 31475571

[B13] Bluhm R. E. Rodgers W. H. Black D. L. Wilkinson G. R. Branch R. (1992). Cholestasis in transplant patients-what is the role of cyclosporin? Aliment. Pharmacol. Ther. 6 (2), 207–219. 10.1111/j.1365-2036.1992.tb00264.x 1600041

[B14] Bramow S. Ott P. Thomsen Nielsen F. Bangert K. Tygstrup N. Dalhoff K. (2001). Cholestasis and regulation of genes related to drug metabolism and biliary transport in rat liver following treatment with cyclosporine A and sirolimus (Rapamycin). Pharmacol. Toxicol. 89 (3), 133–139. 10.1034/j.1600-0773.2001.d01-147.x 11589784

[B15] Bravo-Sagua R. Parra V. Lopez-Crisosto C. Diaz P. Quest A. F. Lavandero S. (2017). Calcium transport and signaling in mitochondria. Compr. Physiol. 7 (2), 623–634. 10.1002/cphy.c160013 28333383

[B16] Broekemeier K. M. Carpenter-Deyo L. Reed D. J. Pfeiffer D. R. (1992). Cyclosporin A protects hepatocytes subjected to high Ca2+ and oxidative stress. FEBS Lett. 304 (2-3), 192–194. 10.1016/0014-5793(92)80616-o 1618322

[B17] Broekemeier K. M. Dempsey M. E. Pfeiffer D. R. (1989). Cyclosporin A is a potent inhibitor of the inner membrane permeability transition in liver mitochondria. J. Biol. Chem. 264 (14), 7826–7830. 10.1016/s0021-9258(18)83116-7 2470734

[B18] Burban A. Sharanek A. Guguen-Guillouzo C. Guillouzo A. (2018). Endoplasmic reticulum stress precedes oxidative stress in antibiotic-induced cholestasis and cytotoxicity in human hepatocytes. Free Radic. Biol. Med. 115, 166–178. 10.1016/j.freeradbiomed.2017.11.017 29191461

[B19] Chan F. K. Shaffer E. A. (1997). Cholestatic effects of cyclosporine in the rat. Transplantation 63 (11), 1574–1578. 10.1097/00007890-199706150-00006 9197348

[B20] Cheriyan A. M. Ume A. C. Francis C. E. King K. N. Linck V. A. Bai Y. (2021). Calcineurin A-α suppression drives nuclear factor-κB-mediated NADPH oxidase-2 upregulation. Am. J. Physiol. Ren. Physiol. 320 (5), F789–F798. 10.1152/ajprenal.00254.2020 PMC8424558 33615888

[B21] Cichoz-Lach H. Michalak A. (2014). Oxidative stress as a crucial factor in liver diseases. World J. Gastroenterol. 20 (25), 8082–8091. 10.3748/wjg.v20.i25.8082 25009380 PMC4081679

[B22] Copple B. L. Jaeschke H. Klaassen C. D. (2010). Oxidative stress and the pathogenesis of cholestasis. Semin. Liver Dis. 30 (2), 195–204. 10.1055/s-0030-1253228 20422501

[B23] Dada F. A. Oyeleye S. I. Adefegha S. A. Babatola L. J. Adebayo A. (2021). Evaluation of different almond (*Terminalia catappa*) extracts against oxidative stress induced by cyclosporine in brain and liver of rats. J. Complement. Integr. Med. 18 (4), 727–735. 10.1515/jcim-2020-0193 33852232

[B24] De Vriese A. S. Coster R. V. Smet J. Seneca S. Lovering A. Van Haute L. L. (2006). Linezolid-induced inhibition of mitochondrial protein synthesis. Clin. Infect. Dis. 42 (8), 1111–1117. 10.1086/501356 16575728

[B25] Demeilliers C. Maisonneuve C. Grodet A. Mansouri A. Nguyen R. Tinel M. (2002). Impaired adaptive resynthesis and prolonged depletion of hepatic mitochondrial DNA after repeated alcohol binges in mice. Gastroenterology 123 (4), 1278–1290. 10.1053/gast.2002.35952 12360488

[B26] Deters M. Strubelt O. Younes M. (1997). Reevaluation of cyclosporine induced hepatotoxicity in the isolated perfused rat liver. Toxicology 123 (3), 197–206. 10.1016/s0300-483x(97)00123-6 9355938

[B27] Farrell G. C. Duddy S. K. Kass G. E. Llopis J. Gahm A. Orrenius S. (1990). Release of Ca2+ from the endoplasmic reticulum is not the mechanism for bile acid-induced cholestasis and hepatotoxicity in the intact rat liver. J. Clin. Invest. 85 (4), 1255–1259. 10.1172/JCI114561 2318979 PMC296560

[B28] Fattizzo B. Cantoni S. Giannotta J. A. Bandiera L. Zavaglia R. Bortolotti M. (2022). Efficacy and safety of cyclosporine A treatment in autoimmune cytopenias: The experience of two Italian reference centers. Ther. Adv. Hematol. 13, 20406207221097780. 10.1177/20406207221097780 35585968 PMC9109490

[B29] Fellman C. L. Archer T. M. Wills R. W. Mackin A. J. (2019). Effects of cyclosporine and dexamethasone on canine T cell expression of interleukin-2 and interferon-gamma. Vet. Immunol. Immunopathol. 216, 109892. 10.1016/j.vetimm.2019.109892 31446206

[B30] Flippin M. S. Canter C. E. Balzer D. T. (2000). Increased morbidity and high variability of cyclosporine levels in pediatric heart transplant recipients. J. Heart Lung Transpl. 19 (4), 343–349. 10.1016/s1053-2498(00)00061-9 10775814

[B31] Forman H. J. Zhang H. Rinna A. (2009). Glutathione: Overview of its protective roles, measurement, and biosynthesis. Mol. Asp. Med. 30 (1-2), 1–12. 10.1016/j.mam.2008.08.006 PMC2696075 18796312

[B32] Fournier N. Ducet G. Crevat A. (1987). Action of cyclosporine on mitochondrial calcium fluxes. J. Bioenerg. Biomembr. 19 (3), 297–303. 10.1007/BF00762419 3114244

[B33] Fromenty B. (2020). Alteration of mitochondrial DNA homeostasis in drug-induced liver injury. Food Chem. Toxicol. 135, 110916. 10.1016/j.fct.2019.110916 31669601

[B34] Fusai G. Dhaliwal P. Rolando N. Sabin C. A. Patch D. Davidson B. R. (2006). Incidence and risk factors for the development of prolonged and severe intrahepatic cholestasis after liver transplantation. Liver Transpl. 12 (11), 1626–1633. 10.1002/lt.20870 16952166

[B35] Garcia-Ruiz C. Fernandez-Checa J. C. (2018). Mitochondrial oxidative stress and antioxidants balance in fatty liver disease. Hepatol. Commun. 2 (12), 1425–1439. 10.1002/hep4.1271 30556032 PMC6287487

[B36] Gijbels E. Vilas-Boas V. Annaert P. Vanhaecke T. Devisscher L. Vinken M. (2020). Robustness testing and optimization of an adverse outcome pathway on cholestatic liver injury. Arch. Toxicol. 94 (4), 1151–1172. 10.1007/s00204-020-02691-9 32152650

[B37] Gores G. J. Miyoshi H. Botla R. Aguilar H. I. Bronk S. F. (1998). Induction of the mitochondrial permeability transition as a mechanism of liver injury during cholestasis: A potential role for mitochondrial proteases. Biochim. Biophys. Acta 1366 (1-2), 167–175. 10.1016/s0005-2728(98)00111-x 9714791

[B38] Greene V. Cao H. Schanne F. A. Bartelt D. C. (2002). Oxidative stress-induced calcium signalling in Aspergillus nidulans. Cell. Signal. 14 (5), 437–443. 10.1016/s0898-6568(01)00266-2 11882388

[B39] Heidari R. Niknahad H. (2019). The role and study of mitochondrial impairment and oxidative stress in cholestasis. Methods Mol. Biol. 1981, 117–132. 10.1007/978-1-4939-9420-5_8 31016651

[B40] Herraiz M. Beraza N. Solano A. Sangro B. Montoya J. Qian C. (2003). Liver failure caused by herpes simplex virus thymidine kinase plus ganciclovir therapy is associated with mitochondrial dysfunction and mitochondrial DNA depletion. Hum. Gene Ther. 14 (5), 463–472. 10.1089/104303403321467225 12691611

[B41] Hicks D. Lampe A. K. Laval S. H. Allamand V. Jimenez-Mallebrera C. Walter M. C. (2009). Cyclosporine A treatment for ullrich congenital muscular dystrophy: A cellular study of mitochondrial dysfunction and its rescue. Brain 132 (1), 147–155. 10.1093/brain/awn289 19015158

[B42] Honrath B. Metz I. Bendridi N. Rieusset J. Culmsee C. Dolga A. M. (2017). Glucose-regulated protein 75 determines ER-mitochondrial coupling and sensitivity to oxidative stress in neuronal cells. Cell Death Discov. 3, 17076. 10.1038/cddiscovery.2017.76 29367884 PMC5672593

[B43] Huang Z. Chen Y. Zhang Y. (2020). Mitochondrial reactive oxygen species cause major oxidative mitochondrial DNA damages and repair pathways. J. Biosci. 45, 84. 10.1007/s12038-020-00055-0 32661211

[B44] Imaizumi N. Aniya Y. (2011). The role of a membrane-bound glutathione transferase in the peroxynitrite-induced mitochondrial permeability transition pore: formation of a disulfide-linked protein complex. Arch. Biochem. Biophys. 516 (2), 160–172. 10.1016/j.abb.2011.10.012 22050912

[B45] Imberti R. Nieminen A. L. Herman B. Lemasters J. J. (1993). Mitochondrial and glycolytic dysfunction in lethal injury to hepatocytes by t-butylhydroperoxide: Protection by fructose, cyclosporin A and trifluoperazine. J. Pharmacol. Exp. Ther. 265 (1), 392–400. 8474021

[B46] Iruzubieta P. Goikoetxea-Usandizaga N. Barbier-Torres L. Serrano-Macia M. Fernandez-Ramos D. Fernandez-Tussy P. (2021). Boosting mitochondria activity by silencing MCJ overcomes cholestasis-induced liver injury. JHEP Rep. 3 (3), 100276. 10.1016/j.jhepr.2021.100276 33997750 PMC8099785

[B47] Jaeschke H. Ramachandran A. (2011). Reactive oxygen species in the normal and acutely injured liver. J. Hepatol. 55 (1), 227–228. 10.1016/j.jhep.2011.01.006 21238521 PMC3117914

[B48] Jimenez R. Galan A. I. Gonzalez de Buitrago J. M. Palomero J. Munoz M. E. (2000). Glutathione metabolism in cyclosporine A-treated rats: Dose- and time-related changes in liver and kidney. Clin. Exp. Pharmacol. Physiol. 27 (12), 991–996. 10.1046/j.1440-1681.2000.03382.x 11117236

[B49] Jung K. Pergande M. (1985). Influence of cyclosporin A on the respiration of isolated rat kidney mitochondria. FEBS Lett. 183 (1), 167–169. 10.1016/0014-5793(85)80977-7 2858411

[B50] Kazuno A. A. Munakata K. Nagai T. Shimozono S. Tanaka M. Yoneda M. (2006). Identification of mitochondrial DNA polymorphisms that alter mitochondrial matrix pH and intracellular calcium dynamics. PLoS Genet. 2 (8), e128. 10.1371/journal.pgen.0020128 16895436 PMC1534079

[B51] Ke H. Huai Q. (2004). Crystal structures of cyclophilin and its partners. Front. Biosci. 9, 2285–2296. 10.2741/1396 15353287

[B52] Kim S. H. Seo H. Kwon D. Yuk D. Y. Jung Y. S. (2022). Taurine ameliorates tunicamycin-induced liver injury by disrupting the vicious cycle between oxidative stress and endoplasmic reticulum stress. Life (Basel) 12 (3), 354. 10.3390/life12030354 35330105 PMC8951380

[B53] Kitamura N. Shindo M. Ohtsuka J. Nakamura A. Tanokura M. Hiroi T. (2020). Identification of novel interacting regions involving calcineurin and nuclear factor of activated T cells. FASEB J. 34 (2), 3197–3208. 10.1096/fj.201902229 31909857

[B54] Klawitter J. Klawitter J. Pennington A. Kirkpatrick B. Roda G. Kotecha N. C. (2019). Cyclophilin D knockout protects the mouse kidney against cyclosporin A-induced oxidative stress. Am. J. Physiol. Ren. Physiol. 317 (3), F683–F694. 10.1152/ajprenal.00417.2018 31188033

[B55] Kolaric T. O. Nincevic V. Smolic R. Smolic M. Wu G. Y. (2019). Mechanisms of hepatic cholestatic drug injury. J. Clin. Transl. Hepatol. 7 (1), 86–92. 10.14218/JCTH.2018.00042 30944824 PMC6441637

[B56] Korolczuk A. Caban K. Amarowicz M. Czechowska G. Irla-Miduch J. (2016). Oxidative stress and liver morphology in experimental cyclosporine A-induced hepatotoxicity. Biomed. Res. Int. 2016, 5823271. 10.1155/2016/5823271 27298826 PMC4889794

[B57] Koziel R. Zablocki K. Duszynski J. (2006). Calcium signals are affected by ciprofloxacin as a consequence of reduction of mitochondrial DNA content in Jurkat cells. Antimicrob. Agents Chemother. 50 (5), 1664–1671. 10.1128/AAC.50.5.1664-1671.2006 16641433 PMC1472211

[B58] Kujoth G. C. Hiona A. Pugh T. D. Someya S. Panzer K. Wohlgemuth S. E. (2005). Mitochondrial DNA mutations, oxidative stress, and apoptosis in mammalian aging. Science 309 (5733), 481–484. 10.1126/science.1112125 16020738

[B59] Lai Q. Luo Z. Wu C. Lai S. Wei H. Li T. (2017). Attenuation of cyclosporine A induced nephrotoxicity by schisandrin B through suppression of oxidative stress, apoptosis and autophagy. Int. Immunopharmacol. 52, 15–23. 10.1016/j.intimp.2017.08.019 28846887

[B60] Larosche I. Letteron P. Fromenty B. Vadrot N. Abbey-Toby A. Feldmann G. (2007). Tamoxifen inhibits topoisomerases, depletes mitochondrial DNA, and triggers steatosis in mouse liver. J. Pharmacol. Exp. Ther. 321 (2), 526–535. 10.1124/jpet.106.114546 17277197

[B61] Lawrence J. W. Darkin-Rattray S. Xie F. Neims A. H. Rowe T. C. (1993). 4-Quinolones cause a selective loss of mitochondrial DNA from mouse L1210 leukemia cells. J. Cell. Biochem. 51 (2), 165–174. 10.1002/jcb.240510208 8440750

[B62] Le Guillou D. Bucher S. Begriche K. Hoet D. Lombes A. Labbe G. (2018). Drug-induced alterations of mitochondrial DNA homeostasis in steatotic and nonsteatotic HepaRG cells. J. Pharmacol. Exp. Ther. 365 (3), 711–726. 10.1124/jpet.117.246751 29669730

[B63] Lee H. C. Wei Y. H. (1997). Mutation and oxidative damage of mitochondrial DNA and defective turnover of mitochondria in human aging. J. Formos. Med. Assoc. 96 (10), 770–778. 9343975

[B64] Lewis W. Levine E. S. Griniuviene B. Tankersley K. O. Colacino J. M. Sommadossi J. P. (1996). Fialuridine and its metabolites inhibit DNA polymerase gamma at sites of multiple adjacent analog incorporation, decrease mtDNA abundance, and cause mitochondrial structural defects in cultured hepatoblasts. Proc. Natl. Acad. Sci. U. S. A. 93 (8), 3592–3597. 10.1073/pnas.93.8.3592 8622980 PMC39655

[B65] Li G. Fu W. Deng Y. Zhao Y. (2021). Role of calcium/calcineurin signalling in regulating intracellular reactive oxygen species homeostasis in *Saccharomyces cerevisiae* . Genes (Basel) 12 (9), 1311. 10.3390/genes12091311 34573294 PMC8466207

[B66] Liu Q. F. Deng Z. Y. Ye J. M. He A. L. Li S. S. (2015). Ginsenoside Rg1 protects chronic cyclosporin a nephropathy from tubular cell apoptosis by inhibiting endoplasmic reticulum stress in rats. Transpl. Proc. 47 (2), 566–569. 10.1016/j.transproceed.2014.10.047 25769608

[B67] Liu R. Xu F. Bi S. Zhao X. Jia B. Cen Y. (2019). Mitochondrial DNA-induced inflammatory responses and lung injury in thermal injury murine model: Protective effect of cyclosporine-A. J. Burn Care Res. 40 (3), 355–360. 10.1093/jbcr/irz029 30926991

[B68] Mansouri A. Haouzi D. Descatoire V. Demeilliers C. Sutton A. Vadrot N. (2003). Tacrine inhibits topoisomerases and DNA synthesis to cause mitochondrial DNA depletion and apoptosis in mouse liver. Hepatology 38 (3), 715–725. 10.1053/jhep.2003.50353 12939598

[B69] Michelucci A. Paolini C. Canato M. Wei-Lapierre L. Pietrangelo L. De Marco A. (2015). Antioxidants protect calsequestrin-1 knockout mice from halothane- and heat-induced sudden death. Anesthesiology 123 (3), 603–617. 10.1097/ALN.0000000000000748 26132720 PMC4543432

[B70] Mironova G. D. Pavlov E. V. (2021). Mitochondrial cyclosporine A-independent palmitate/Ca(2+)-induced permeability transition pore (PA-mPT pore) and its role in mitochondrial function and protection against calcium overload and glutamate toxicity. Cells 10 (1), 125. 10.3390/cells10010125 33440765 PMC7827677

[B71] Mohammad Mehdi O. H. Attari H. Siavashpour A. Shafaghat M. Azarpira N. Ghaffari H. (2021). Mitigation of cholestasis-associated hepatic and renal injury by edaravone treatment: Evaluation of its effects on oxidative stress and mitochondrial function. Liver Res. 5 (3), 181–193. 10.1016/j.livres.2020.10.003 PMC11791843 39957848

[B72] Moran D. De Buitrago J. M. Fernandez E. Galan A. I. Munoz M. E. Jimenez R. (1998). Inhibition of biliary glutathione secretion by cyclosporine A in the rat: Possible mechanisms and role in the cholestasis induced by the drug. J. Hepatol. 29 (1), 68–77. 10.1016/s0168-8278(98)80180-3 9696494

[B73] Nguyen K. D. Sundaram V. Ayoub W. S. (2014). Atypical causes of cholestasis. World J. Gastroenterol. 20 (28), 9418–9426. 10.3748/wjg.v20.i28.9418 25071336 PMC4110573

[B74] Nicotera P. McConkey D. Svensson S. A. Bellomo G. Orrenius S. (1988). Correlation between cytosolic Ca2+ concentration and cytotoxicity in hepatocytes exposed to oxidative stress. Toxicology 52 (1-2), 55–63. 10.1016/0300-483x(88)90196-5 3188034

[B75] Nissanka N. Moraes C. T. (2018). Mitochondrial DNA damage and reactive oxygen species in neurodegenerative disease. FEBS Lett. 592 (5), 728–742. 10.1002/1873-3468.12956 29281123 PMC6942696

[B76] Oliva-Vilarnau N. Hankeova S. Vorrink S. U. Mkrtchian S. Andersson E. R. Lauschke V. M. (2018). Calcium signaling in liver injury and regeneration. Front. Med. 5, 192. 10.3389/fmed.2018.00192 PMC6039545 30023358

[B77] Pallet N. Bouvier N. Bendjallabah A. Rabant M. Flinois J. P. Hertig A. (2008). Cyclosporine-induced endoplasmic reticulum stress triggers tubular phenotypic changes and death. Am. J. Transpl. 8 (11), 2283–2296. 10.1111/j.1600-6143.2008.02396.x 18785955

[B78] Palomero J. Galan A. I. Munoz M. E. Tunon M. J. Gonzalez-Gallego J. Jimenez R. (2001). Effects of aging on the susceptibility to the toxic effects of cyclosporin A in rats. Changes in liver glutathione and antioxidant enzymes. Free Radic. Biol. Med. 30 (8), 836–845. 10.1016/s0891-5849(01)00471-3 11295526

[B79] Paolini C. Quarta M. Wei-LaPierre L. Michelucci A. Nori A. Reggiani C. (2015). Oxidative stress, mitochondrial damage, and cores in muscle from calsequestrin-1 knockout mice. Skelet. Muscle 5, 10. 10.1186/s13395-015-0035-9 26075051 PMC4464246

[B80] Pastore A. Federici G. Bertini E. Piemonte F. (2003). Analysis of glutathione: Implication in redox and detoxification. Clin. Chim. Acta. 333 (1), 19–39. 10.1016/s0009-8981(03)00200-6 12809732

[B81] Patocka J. Nepovimova E. Kuca K. Wu W. (2021). Cyclosporine A: Chemistry and toxicity - a review. Curr. Med. Chem. 28 (20), 3925–3934. 10.2174/0929867327666201006153202 33023428

[B82] Pecorella I. Ciardi A. Rahimi S. Di Tondo U. Di Tondo U. (1990). Cholestasis in liver transplantation: Incidence and diagnostic significance. Pathologica 82 (1081), 513–518. 2080094

[B83] Peng T. I. Jou M. J. (2010). Oxidative stress caused by mitochondrial calcium overload. Ann. N. Y. Acad. Sci. 1201, 183–188. 10.1111/j.1749-6632.2010.05634.x 20649555

[B84] Qin H. Zhang L. L. Xiong X. L. Jiang Z. X. Xiao C. P. Zhang L. L. (2020). Li-Dan-He-Ji improves infantile cholestasis hepatopathy through inhibiting calcium-sensing receptor-mediated hepatocyte apoptosis. Front. Pharmacol. 11, 156. 10.3389/fphar.2020.00156 32180721 PMC7059769

[B85] Rehman H. Ramshesh V. K. Theruvath T. P. Kim I. Currin R. T. Giri S. (2008). NIM811 (N-methyl-4-isoleucine cyclosporine), a mitochondrial permeability transition inhibitor, attenuates cholestatic liver injury but not fibrosis in mice. J. Pharmacol. Exp. Ther. 327 (3), 699–706. 10.1124/jpet.108.143578 18801946 PMC2582973

[B86] Reichen J. Berr F. Le M. Warren G. H. (1985). Characterization of calcium deprivation-induced cholestasis in the perfused rat liver. Am. J. Physiol. 249 (1), G48–G57. 10.1152/ajpgi.1985.249.1.G48 3893157

[B87] Reuter S. Gupta S. C. Chaturvedi M. M. Aggarwal B. B. (2010). Oxidative stress, inflammation, and cancer: How are they linked? Free Radic. Biol. Med. 49 (11), 1603–1616. 10.1016/j.freeradbiomed.2010.09.006 20840865 PMC2990475

[B88] Rezzani R. (2004). Cyclosporine A and adverse effects on organs: Histochemical studies. Prog. Histochem. Cytochem. 39 (2), 85–128. 10.1016/j.proghi.2004.04.001 15354618

[B89] Rodrigues-Diez R. Gonzalez-Guerrero C. Ocana-Salceda C. Rodrigues-Diez R. R. Egido J. Ortiz A. (2016). Calcineurin inhibitors cyclosporine A and tacrolimus induce vascular inflammation and endothelial activation through TLR4 signaling. Sci. Rep. 6, 27915. 10.1038/srep27915 27295076 PMC4904742

[B90] Rolo A. P. Oliveira P. J. Moreno A. J. Palmeira C. M. (2000). Bile acids affect liver mitochondrial bioenergetics: Possible relevance for cholestasis therapy. Toxicol. Sci. 57 (1), 177–185. 10.1093/toxsci/57.1.177 10966524

[B91] Salducci M. D. Chauvet-Monges A. M. Tillement J. P. Albengres E. Testa B. Carrupt P. (1996). Trimetazidine reverses calcium accumulation and impairment of phosphorylation induced by cyclosporine A in isolated rat liver mitochondria. J. Pharmacol. Exp. Ther. 277 (1), 417–422. 8613950

[B92] Samra N. M. Emad El Abrak S. El Dash H. H. El Said El Raziky M. El Sheikh M. A. (2018). Evaluation of vitamin D status bone mineral density and dental health in children with cholestasis. Clin. Res. Hepatol. Gastroenterol. 42 (4), 368–377. 10.1016/j.clinre.2017.11.010 29551613

[B93] Santamaria E. Avila M. A. Latasa M. U. Rubio A. Martin-Duce A. Lu S. C. (2003). Functional proteomics of nonalcoholic steatohepatitis: Mitochondrial proteins as targets of S-adenosylmethionine. Proc. Natl. Acad. Sci. U. S. A. 100 (6), 3065–3070. 10.1073/pnas.0536625100 12631701 PMC152247

[B94] Sasaki M. Yoshimura-Miyakoshi M. Sato Y. Nakanuma Y. (2015). A possible involvement of endoplasmic reticulum stress in biliary epithelial autophagy and senescence in primary biliary cirrhosis. J. Gastroenterol. 50 (9), 984–995. 10.1007/s00535-014-1033-0 25552342

[B95] Schmeits P. C. Schaap M. M. Luijten M. van Someren E. Boorsma A. van Loveren H. (2015). Detection of the mechanism of immunotoxicity of cyclosporine A in murine *in vitro* and *in vivo* models. Arch. Toxicol. 89 (12), 2325–2337. 10.1007/s00204-014-1365-9 25224403

[B96] Sharanek A. Azzi P. B. Al-Attrache H. Savary C. C. Humbert L. Rainteau D. (2014). Different dose-dependent mechanisms are involved in early cyclosporine a-induced cholestatic effects in hepaRG cells. Toxicol. Sci. 141 (1), 244–253. 10.1093/toxsci/kfu122 24973091 PMC4833109

[B97] Sherer T. B. Trimmer P. A. Parks J. K. Tuttle J. B. (2000). Mitochondrial DNA-depleted neuroblastoma (Rho degrees) cells exhibit altered calcium signaling. Biochim. Biophys. Acta 1496 (2-3), 341–355. 10.1016/s0167-4889(00)00027-6 10771102

[B98] Shibao K. Hirata K. Robert M. E. Nathanson M. H. (2003). Loss of inositol 1, 4, 5-trisphosphate receptors from bile duct epithelia is a common event in cholestasis. Gastroenterology 125 (4), 1175–1187. 10.1016/s0016-5085(03)01201-0 14517800 PMC2831084

[B99] Skinner S. K. Solania A. Wolan D. W. Cohen M. S. Ryan T. E. Hepple R. T. (2021). Mitochondrial permeability transition causes mitochondrial reactive oxygen species- and caspase 3-dependent atrophy of single adult mouse skeletal muscle fibers. Cells 10 (10), 2586. 10.3390/cells10102586 34685566 PMC8534155

[B100] Skrtic M. Sriskanthadevan S. Jhas B. Gebbia M. Wang X. Wang Z. (2011). Inhibition of mitochondrial translation as a therapeutic strategy for human acute myeloid leukemia. Cancer Cell 20 (5), 674–688. 10.1016/j.ccr.2011.10.015 22094260 PMC3221282

[B101] Sokol R. J. Dahl R. Devereaux M. W. Yerushalmi B. Kobak G. E. Gumpricht E. (2005). Human hepatic mitochondria generate reactive oxygen species and undergo the permeability transition in response to hydrophobic bile acids. J. Pediatr. Gastroenterol. Nutr. 41 (2), 235–243. 10.1097/01.mpg.0000170600.80640.88 16056106

[B102] Soresi M. Sparacino V. Pisciotta G. Bonfissuto G. Caputo F. Carroccio A. (1995). Effects of cyclosporin A on various indices of cholestasis in kidney transplant recipients. Minerva Urol. Nefrol. 47 (2), 65–69. 8560351

[B103] Stacy Grieve D. B. Biswas D. (2022). “Targeting reactive oxygen species homeostasis and metabolism in cancer stem cells,” in Handbook of oxidative stress in cancer: Mechanistic aspects, 2385–2405. 10.1007/978-981-15-9411-3_150

[B104] Taniai N. Akimaru K. Ishikawa Y. Kanada T. Kakinuma D. Mizuguchi Y. (2008). Hepatotoxicity caused by both tacrolimus and cyclosporine after living donor liver transplantation. J. Nippon. Med. Sch. 75 (3), 187–191. 10.1272/jnms.75.187 18648179

[B105] Tiao M. M. Lin T. K. Liou C. W. Wang P. W. Chen J. B. Kuo F. Y. (2009). Early transcriptional deregulation of hepatic mitochondrial biogenesis and its consequent effects on murine cholestatic liver injury. Apoptosis 14 (7), 890–899. 10.1007/s10495-009-0357-3 19462240

[B106] Vickers A. E. Ulyanov A. V. Fisher R. L. (2017). Liver effects of clinical drugs differentiated in human liver slices. Int. J. Mol. Sci. 18 (3), E574. 10.3390/ijms18030574 28272341 PMC5372590

[B107] Vinken M. (2015). Adverse outcome pathways and drug-induced liver injury testing. Chem. Res. Toxicol. 28 (7), 1391–1397. 10.1021/acs.chemrestox.5b00208 26119269 PMC4596002

[B108] Visentin M. Lenggenhager D. Gai Z. Kullak-Ublick G. A. (2018). Drug-induced bile duct injury. Biochim. Biophys. Acta. Mol. Basis Dis. 1864 (4), 1498–1506. 10.1016/j.bbadis.2017.08.033 28882625

[B109] Wagner S. Seidler T. Picht E. Maier L. S. Kazanski V. Teucher N. (2003). Na(+)-Ca(2+) exchanger overexpression predisposes to reactive oxygen species-induced injury. Cardiovasc. Res. 60 (2), 404–412. 10.1016/j.cardiores.2003.08.006 14613870

[B110] Wang J. He W. Tsai P. J. Chen P. H. Ye M. Guo J. (2020). Mutual interaction between endoplasmic reticulum and mitochondria in nonalcoholic fatty liver disease. Lipids Health Dis. 19 (1), 72. 10.1186/s12944-020-01210-0 32284046 PMC7155254

[B111] West A. P. Brodsky I. E. Rahner C. Woo D. K. Erdjument-Bromage H. Tempst P. (2011). TLR signalling augments macrophage bactericidal activity through mitochondrial ROS. Nature 472 (7344), 476–480. 10.1038/nature09973 21525932 PMC3460538

[B112] Wolf A. Trendelenburg C. F. Diez-Fernandez C. Prieto P. Houy S. Trommer W. E. (1997). Cyclosporine A-induced oxidative stress in rat hepatocytes. J. Pharmacol. Exp. Ther. 280 (3), 1328–1334. 9067320

[B113] Wolters J. E. J. van Breda S. G. J. Caiment F. Claessen S. M. de Kok T. Kleinjans J. C. S. (2017). Nuclear and mitochondrial DNA methylation patterns induced by valproic acid in human hepatocytes. Chem. Res. Toxicol. 30 (10), 1847–1854. 10.1021/acs.chemrestox.7b00171 28853863 PMC5645762

[B114] Xiao W. Loscalzo J. (2020). Metabolic responses to reductive stress. Antioxid. Redox Signal. 32 (18), 1330–1347. 10.1089/ars.2019.7803 31218894 PMC7247050

[B115] Xiao Z. Jia B. Zhao X. Bi S. Meng W. (2018). Attenuation of lipopolysaccharide-induced acute lung injury by cyclosporine-A via suppression of mitochondrial DNA. Med. Sci. Monit. 24, 7682–7688. 10.12659/MSM.909909 30367813 PMC6216435

[B116] Xu S. C. Chen Y. B. Lin H. Pi H. F. Zhang N. X. Zhao C. C. (2012). Damage to mtDNA in liver injury of patients with extrahepatic cholestasis: The protective effects of mitochondrial transcription factor A. Free Radic. Biol. Med. 52 (9), 1543–1551. 10.1016/j.freeradbiomed.2012.01.007 22306509

[B117] Yalcin K. Celen S. Zhumatayev S. Daloglu H. Pashayev D. Ozturkmen S. (2021). Analyzing the clinical outcomes of switching from cyclosporine to tacrolimus in pediatric hematopoietic stem cell transplantation. Clin. Transpl. 35 (7), e14328. 10.1111/ctr.14328 33896035

[B118] Yang W. Tao Y. Wu Y. Zhao X. Ye W. Zhao D. (2019). Neutrophils promote the development of reparative macrophages mediated by ROS to orchestrate liver repair. Nat. Commun. 10 (1), 1076. 10.1038/s41467-019-09046-8 30842418 PMC6403250

[B119] Yerushalmi B. Dahl R. Devereaux M. W. Gumpricht E. Sokol R. J. (2001). Bile acid-induced rat hepatocyte apoptosis is inhibited by antioxidants and blockers of the mitochondrial permeability transition. Hepatology 33 (3), 616–626. 10.1053/jhep.2001.22702 11230742

[B120] Zeeshan H. M. Lee G. H. Kim H. R. Chae H. J. (2016). Endoplasmic reticulum stress and associated ROS. Int. J. Mol. Sci. 17 (3), 327. 10.3390/ijms17030327 26950115 PMC4813189

[B121] Zhang T. Guo J. Gu J. Chen K. Li H. Wang J. (2019). Protective role of mTOR in liver ischemia/reperfusion injury: Involvement of inflammation and autophagy. Oxid. Med. Cell. Longev. 2019, 7861290. 10.1155/2019/7861290 31827701 PMC6885218

[B122] Zhang X. Wu X. Hu Q. Wu J. Wang G. Hong Z. (2019). Mitochondrial DNA in liver inflammation and oxidative stress. Life Sci. 236, 116464. 10.1016/j.lfs.2019.05.020 31078546

[B123] Zhao M. Wang Y. Li L. Liu S. Wang C. Yuan Y. (2021). Mitochondrial ROS promote mitochondrial dysfunction and inflammation in ischemic acute kidney injury by disrupting TFAM-mediated mtDNA maintenance. Theranostics 11 (4), 1845–1863. 10.7150/thno.50905 33408785 PMC7778599

[B124] Zhou A. Y. Ryeom S. (2014). Cyclosporin A promotes tumor angiogenesis in a calcineurin-independent manner by increasing mitochondrial reactive oxygen species. Mol. Cancer Res. 12 (11), 1663–1676. 10.1158/1541-7786.MCR-14-0136 25009293 PMC4233164

[B125] Zhu J. Dong X. Liu Q. Wu C. Wang Q. Long Z. (2016). Hydrophobic bile acids relax rat detrusor contraction via inhibiting the opening of the Na⁺/Ca²⁺ exchanger. Sci. Rep. 6, 21358. 10.1038/srep21358 26892434 PMC4759538

[B126] Zu Y. Wan L. J. Cui S. Y. Gong Y. P. Li C. L. (2015). The mitochondrial Na(+)/Ca(2+) exchanger may reduce high glucose-induced oxidative stress and nucleotide-binding oligomerization domain receptor 3 inflammasome activation in endothelial cells. J. Geriatr. Cardiol. 12 (3), 270–278. 10.11909/j.issn.1671-5411.2015.03.003 26089852 PMC4460171

